# Supplementary material for: Feedback control of heart rate during treadmill exercise based on a two-phase response model
Source: PLoS One. 2023 Oct 23;18(10):e0292310. doi: 10.1371/journal.pone.0292310 (PMC10593204; doi:10.1371/journal.pone.0292310)
Supplement: Attachment — Submitted filename: Response to Reviewers.docx [file pone.0292310.s001.docx]

**Editor**

**RESPONSE:** Thank you for your constructive and helpful comments. Please find our point-by-point responses below..

3. Please update your submission to use the PLOS LaTeX template. The template and more information on our requirements for LaTeX submissions can be found at <http://journals.plos.org/plosone/s/latex>.

**RESPONSE**: The template has been applied.

5. Your ethics statement should only appear in the Methods section of your manuscript. If your ethics statement is written in any section besides the Methods, please delete it from any other section.

**RESPONSE**: The ethics statement was moved to the Methods section.

6. We note that Figure 4 includes an image of a participant in the study.

As per the PLOS ONE policy (http://journals.plos.org/plosone/s/submission-guidelines#loc-human-subjects-research) on papers that include identifying, or potentially identifying, information, the individual(s) or parent(s)/guardian(s) must be informed of the terms of the PLOS open-access (CC-BY) license and provide specific permission for publication of these details under the terms of this license. Please download the Consent Form for Publication in a PLOS Journal (http://journals.plos.org/plosone/s/file?id=8ce6/plos-consent-form-english.pdf). The signed consent form should not be submitted with the manuscript, but should be securely filed in the individual's case notes. Please amend the methods section and ethics statement of the manuscript to explicitly state that the patient/participant has provided consent for publication: “The individual in this manuscript has given written informed consent (as outlined in PLOS consent form) to publish these case details”.

If you are unable to obtain consent from the subject of the photograph, you will need to remove the figure and any other textual identifying information or case descriptions for this individual.

**RESPONSE**: The person in Fig. 4 is not a study participant, rather the part of this figure showing the treadmill and runner was provided to us with explicit approval from the treadmill manufacturer h/p/cosmos – this forms part of their product literature. To prevent identification of this person, their face has now been blurred.

7. Please review your reference list to ensure that it is complete and correct. If you have cited papers that have been retracted, please include the rationale for doing so in the manuscript text, or remove these references and replace them with relevant current references. Any changes to the reference list should be mentioned in the rebuttal letter that accompanies your revised manuscript. If you need to cite a retracted article, indicate the article’s retracted status in the References list and also include a citation and full reference for the retraction notice.

**RESPONSE**: The reference list has been reviewed as instructed.

**Reviewer #1**

**RESPONSE:** Thank you for your constructive and helpful comments. Please find below a point-by-point response. Changes to the manuscript have been highlighted in red font.

1) In the abstract, there are some artifacts, due to an incorrect syntax in referencing to some equations. This should be fixed.

**RESPONSE:**

This has been corrected.

2) The authors say that the controller based on a second-order model (C2) is more "dynamic" with respect to the one based on a first-order model (C1). It is not clear what the authors mean with the term "dynamic". Furthermore, only in the "Experimental results" it is stated that it is related to the control u having an higher "average power of change". This is not clear.

**RESPONSE:**

To clarify this point, the following text has been added to the Introduction (lines 79-82):

“The concept of a controller being more or less dynamic is quantified in this work using the average power of changes in the control signal (the treadmill speed command; see Eq. (32) in the sequel).”

This outcome, the average power of changes in the control signal, was already explicitly defined in the Methods, viz. in Eq. (32).

3) In the "Results" section, there are missing references (e.g. Tab. 1, Fig. ???). These error should be fixed.

**RESPONSE:**

This has been corrected.

4) Figure 5 could use better colors/styles for the signals. The lines overlap too much and it is not clear why the authors have used the red color for both the "nominal evaluation period for outcome calculation" and to represent the measured HR. Furthermore, the legend is wrong, because in the text there is no reference to the "HR_{nom}" signal, while it is mentioned a "HR_{sim}".

**RESPONSE:**

1. We have changed the colour of the horizontal bar indicating the evaluation period to GREEN, to avoid any conflict with the red colour used for HR.
2. Given that the controller aims to ensure that the measured HR follows the target and nominal/simulated HR, and since the controllers in this work are very accurate, it is inevitable that there will be signal overlap. To make things clearer, however, we have replotted these graphs to bring the red lines for measured HR to the forefront of the plots.
3. The legends have all been corrected to use only “sim” as in the corresponding Eq. (31).

5) Although I suppose it would have been difficult to achieve, I think that a direct comparison between the performances obtained with the controllers C1 and C2 (e.g. showing both the use of controller C1 and C2 for the same participant), would have been more useful, to better highlight the performance improvement that a second-order controller has over a first-order one.

**RESPONSE:**

We have added a new figure – Fig. 7 – that shows a direct comparison of C1 and C2 for the same participant – participant P10. As suggested, this highlights the performance improvement for C2 vs. C1. The following text has been added to the Results (lines 285-286):

“To better demonstrate the performance improvement of C2, a pair of measurements from participant P10 are provided (Fig 7).”
